# Supplementary figures and images for: Conserved nuclear hormone receptors controlling a novel plastic trait target fast-evolving genes expressed in a single cell
Source: PLoS Genet. 2020 Apr 13;16(4):e1008687. doi: 10.1371/journal.pgen.1008687 (PMC7179942; doi:10.1371/journal.pgen.1008687)

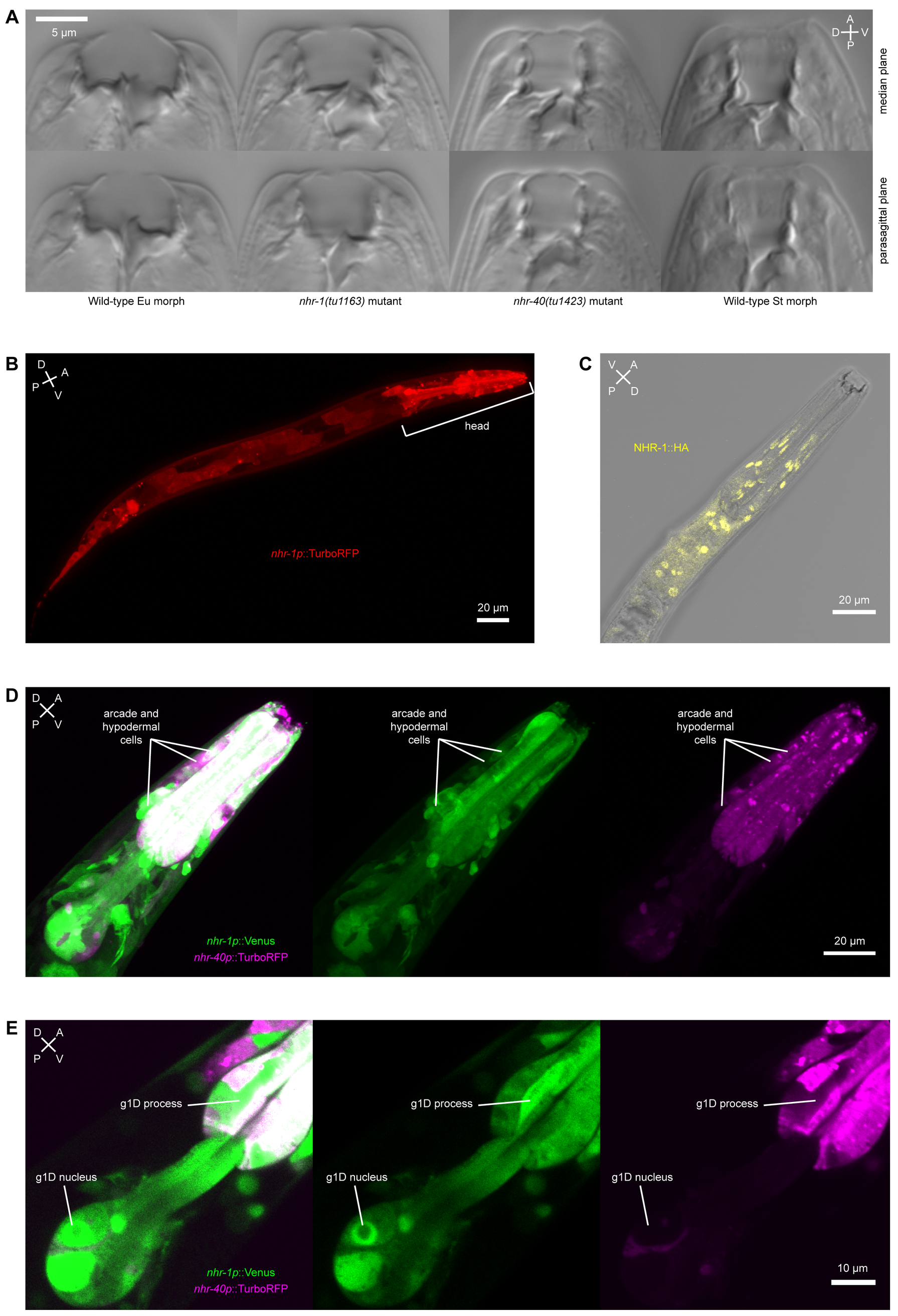

Supplement: S1 Fig — (A) The mouth of the wild-type eurystomatous (Eu) morph, wild-type stenostomatous (St) morph, nhr-1 mutant, and nhr-40 mutant in two focal planes. (B) Expression pattern of an nhr-1 transcriptional reporter in a young larva. TurboRFP channel is presented as a maximum intensity projection. (C) Antibody staining against the HA epitope in a line, in which the tag was “knocked in” into the endogenous locus. Fluorescent channel is presented as a maximum intensity projection. (D) Expression patterns of nhr-40 and nhr-1 transcriptional reporters in a double reporter line. TurboRFP (magenta) and Venus (green) channels are presented as standard deviation and maximum intensity projections, respectively. Co-expression results in white color. (E) Expression patterns of nhr-40 and nhr-1 transcriptional reporters in a double reporter line. TurboRFP is encoded as magenta, Venus as green. Co-expression results in white color. D = dorsal, V = ventral, A = anterior, P = posterior. (TIF) [file pgen.1008687.s001.tif]

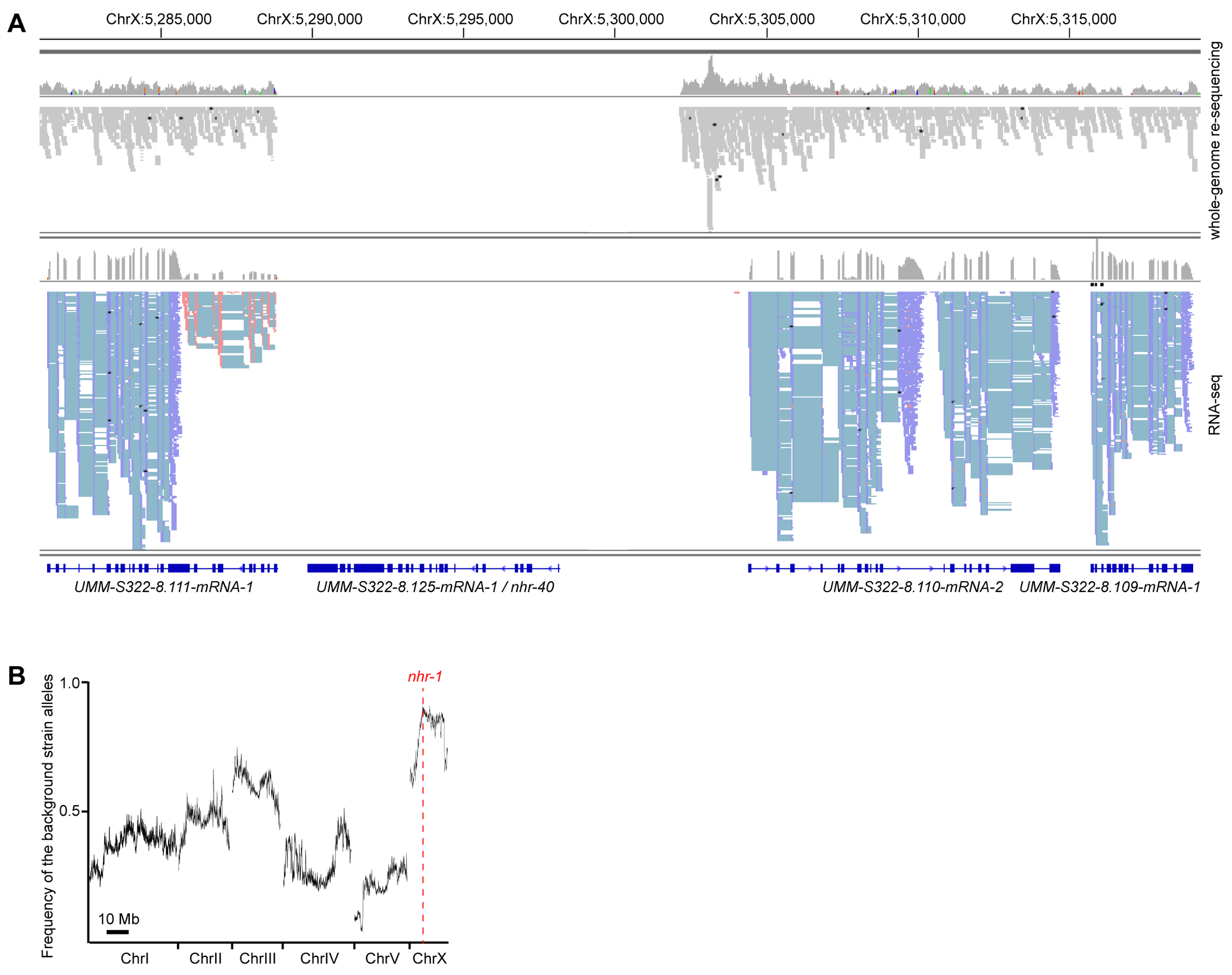

Supplement: S2 Fig — (A) Whole-genome re-sequencing and RNA-seq of the null allele of nhr-40. (B) bulked segregant analysis of the suppressor of nhr-40(tu505) with the location of nhr-1 marked with a dotted line. See S1 Table for the list of non-synonymous and nonsense substitutions within the candidate region. (TIF) [file pgen.1008687.s002.tif]

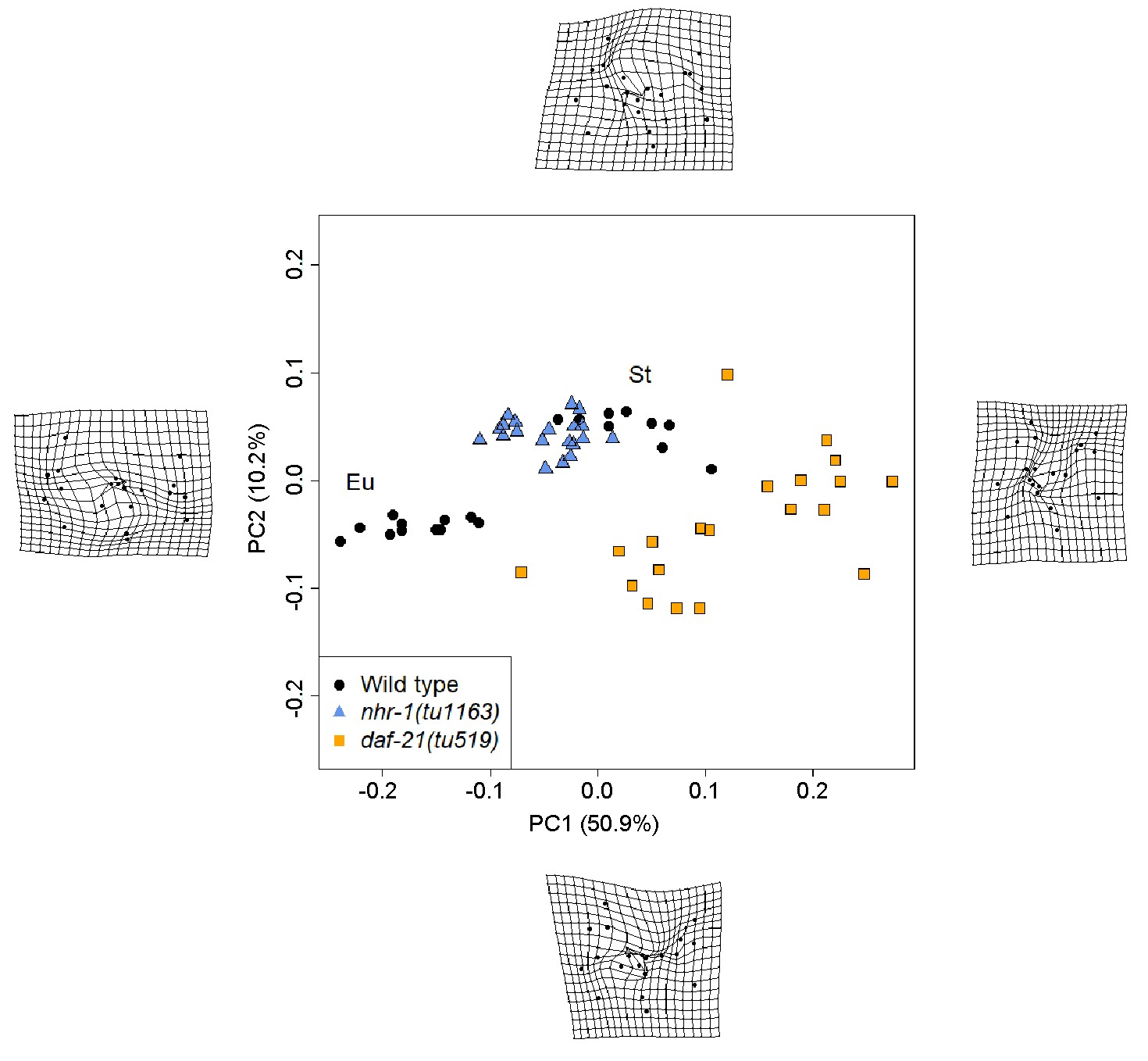

Supplement: S3 Fig — Geometric morphometric analysis of 20 landmarks in the mouth of the wild-type strain RS2333, the nhr-1(tu1163) mutant isolated in this study, and the daf-21/Hsp90(tu519) mutant previously shown to exhibit an aberrant mouth morphology while maintaining the dimorphism. Each point in the PCA plot corresponds to a single animal. Deformation grids at the extremes of the two axes display differences to the mean shape of all individuals. (TIF) [file pgen.1008687.s003.tif]

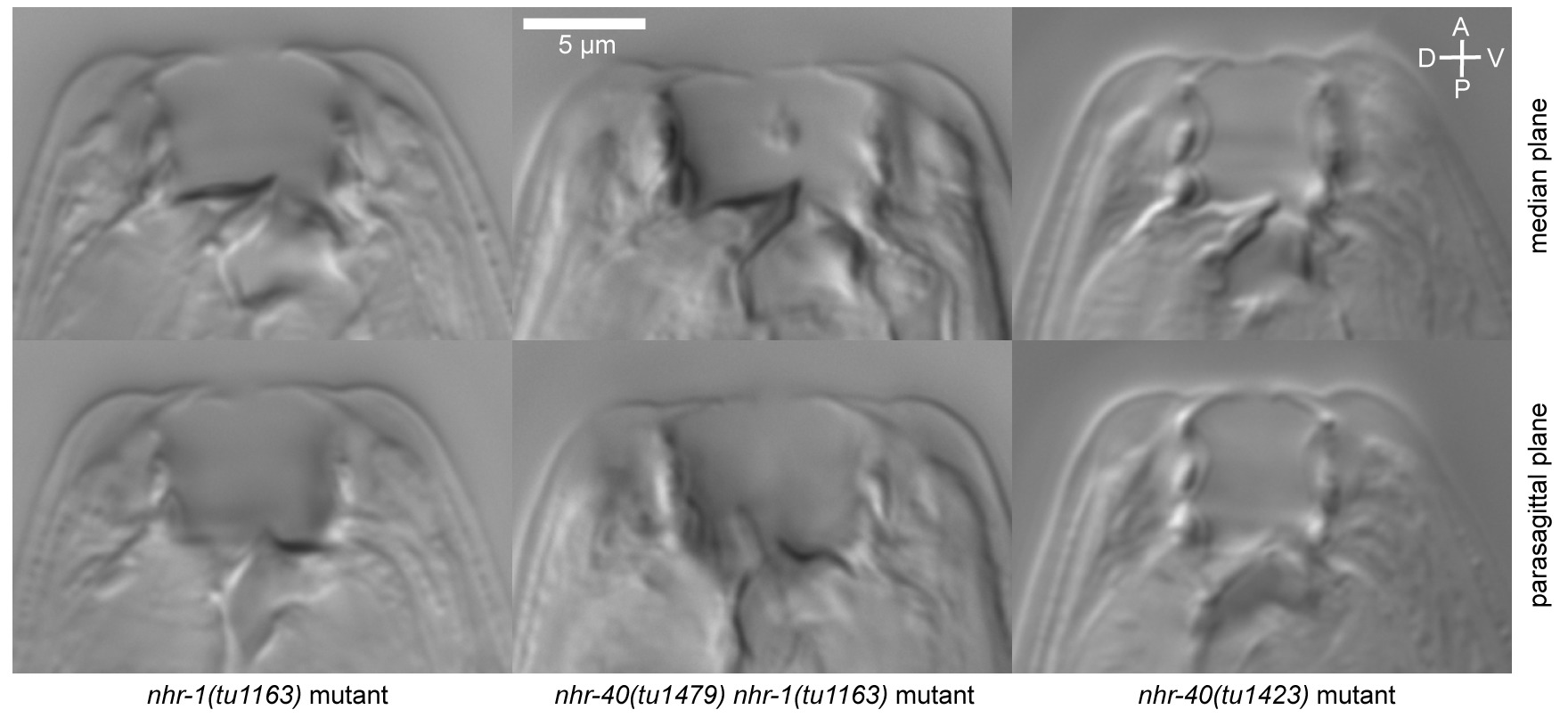

Supplement: S4 Fig — The mouth of the nhr-1 loss-of-function mutant, nhr-40 null mutant and the double nhr-40 loss-of-function nhr-1 loss-of-function mutant in two focal planes. D = dorsal, V = ventral, A = anterior, P = posterior. (TIF) [file pgen.1008687.s004.tif]
