# Supplementary material for: Conserved nuclear hormone receptors controlling a novel plastic trait target fast-evolving genes expressed in a single cell
Source: PLoS Genet. 2020 Apr 13;16(4):e1008687. doi: 10.1371/journal.pgen.1008687 (PMC7179942; doi:10.1371/journal.pgen.1008687)
Supplement: Attachment — Submitted filename: Review.pdf [file pgen.1008687.s012.pdf]

Review of "Conserved hormone-receptors controlling a novel plastic trait target fast-evolving genes expressed in a single cell"

Summary

Developmental plasticity is a topic of broad interest to both developmental and evolutionary biologists. One of the leading models for studying plasticity involves the two possible mouth morphs of the nematode *Pristionchus pacificus.* Sometimes these animals develop a smaller mouth best suited for eating bacteria, known as the Stenostomatous form, whereas in other conditions they develop a sharp-toothed predatory mouth, know as the Eurystomatous form. In this manuscript, the authors dissected the genetic control of these morphs. (1) They screened for suppressors of the *nhr-40(tu505)* mutation, which causes all animals to become Eurystomatous, and recovered null alleles of the nuclear hormone receptor *nhr-1.* These mutations result in animals that develop mouths with an intermediate phenotype. (2) They showed that the *nhr-40(tu505)* allele causes a gain of function, and that null alleles of this gene have the opposite effect — they cause all animals to become Stenostomatous. (3) They found that mutations in *nhr-1* or *nhr-40* have no effect on each other's expression, at least at a whole-animal level. (4) They identified a set of genes whose transcription appears to be regulated by both *nhr-1* and *nhr-40,* and found that these are mainly secreted proteins, that those tested are expressed in the pharyngeal gland cell g1D, which might be involved in mouth formation, and that they are highly redundant. (5) They showed that NHR-1 and NHR-40 are evolving relatively slowly, whereas their targets are largely new genes created by duplication and divergence. Taken together, their data suggest that developmental plastic regulatory networks can evolve through the enlistment of rapidly evolving genes by conserved regulators.

Recommendation

Accept after minor revisions

Comments

(1) The authors raise the question of NHR-1 and NHR-40 perhaps working as a heterodimer, but do not address it further. Do they feel that the phenotypic differences between the two null mutants are large enough to preclude this possibility? If so, they should make the argument clearly. Have they tested interaction in the yeast two-hybrid system, or in another manner? If so, even experiments which do not completely resolve the question would be good to include here.

(2) Although the authors clearly demonstrate that *nhr-1* null mutations are epistatic to *nhr-40(gf)* mutations, they do not appear to have tested whether they are also epistatic to *nhr-40(null)* alleles. This test should be simple and is an important way of probing the relationship between the two genes, and of further testing the model that *nhr-1* mutants are the most downstream because they are involved in cell differentiation, rather than cell fate specification.

(3) The analysis of potential targets of *nhr-1* and *nhr-40* forms a critical part of the paper, and is largely solid. However, a few issues could be cleared up. First, it would be helpful for them to emphasize clearly throughout that the targets could be direct or indirect. Second, have they tested

any of the transcriptional reporters in *nhr-1* or *nhr-40* backgrounds? Looking at a few of these could give a clearer impression of cell-by-cell regulation, as opposed to the whole body regulation of the RNAseq studies.

(4) Perhaps most important, the knockouts of potential target genes were beautifully done, but the lack of phenotypes is surprising. Have the authors considered ablating the cell they implicate through the expression studies, g1D? This might be a quick and easy test to confirm that the central topic of half their paper is indeed involved in controlling mouth morph development.

(5) The extreme redundancy the authors observe can also be problematic from an evolutionary perspective, and they should expand their discussion to address this point.

(6) Although we like the author's evolutionary model at the end, they state it a little too strongly. For example, the sentences: "We speculate that the striking co-expression of the target genes results from an ancient regulatory linkage between the NHRs and the promoters of the ancestral target genes. Such divergent evolutionary dynamics of transcription factors and their downstream targets might represent general features of GRNs" This summary neglects the possibility of these transcription factors capturing new promoters by mutation of target sites. Do we have any information about how complex NHR-1 or NHR-40 target sequences might be?

(7) Since all of the target genes are expressed in the pharyngeal gland cell, g1D, which has a long process that terminates into the buccal cavity, is it possible that the NHR target genes are involved in sensing environmental factors. For example, is the duodecuple Astacin mutant less sensitive to environmental signals.

Minor Changes

Figure 2	FPKM should be defined in the legend
Line 98	Change "and we are yet to find" to "and we had yet to find"
Line 254	Change "NHR-40 and NHR-1 where more highly expressed" to "NHR-40 and NHR-1 were more highly expressed"
Line 390	Change "On the contrary, we identified" to "By contrast, we showed "
