# Supplementary material for: Conserved nuclear hormone receptors controlling a novel plastic trait target fast-evolving genes expressed in a single cell
Source: PLoS Genet. 2020 Apr 13;16(4):e1008687. doi: 10.1371/journal.pgen.1008687 (PMC7179942; doi:10.1371/journal.pgen.1008687)
Supplement: Attachment — Submitted filename: point-by-point.docx [file pgen.1008687.s013.docx]

Dear Editors,

Thank you very much for your email with the positive evaluation of our manuscript. We were very pleased to see the overall evaluation. Please see our point-by-point response below:

**Reviewer #1:**

I have only two small suggestions:

Page 9: “the lack of linear transcriptional regulation is consistent with different phenotypic effects of nhr-1 and nhr-40” – this is only a reasonable statement if one NHR would transcriptionally activate the other, not when it would repress. Remove or rephrase.

We removed this sentence.

Page 13: the discussion on X-chromosome location of NHR target genes may be more appropriate in the Discussion of the paper.

We moved the discussion of the X-chromosomal location to the Discussion section.

**Reviewer #2:**

(1) The authors raise the question of NHR-1 and NHR-40 perhaps working as a heterodimer, but do not address it further. Do they feel that the phenotypic differences between the two null mutants are large enough to preclude this possibility? If so, they should make the argument clearly. Have they tested interaction in the yeast two-hybrid system, or in another manner? If so, even experiments which do not completely resolve the question would be good to include here.

We felt that the phenotypic differences between the null mutants does not support the formation of heterodimers, but it also does not completely rule this out. Therefore, we attempted to perform IP experiments with the FLAG- and HA-tagged versions of NHR-1, but faced multiple challenges, which may have been caused by relatively low expression levels of *nhr-1* or insufficient sensitivity of the tested antibodies in Western Blots. Under these circumstances, we felt that it would be premature to make any statement in the manuscript.

(2) Although the authors clearly demonstrate that nhr-1 null mutations are epistatic to nhr-40(gf) mutations, they do not appear to have tested whether they are also epistatic to nhr-40(null) alleles. This test should be simple and is an important way of probing the relationship between the two genes, and of further testing the model that nhr-1 mutants are the most downstream because they are involved in cell differentiation, rather than cell fate specification.

This is a great suggestion. We had performed this experiment during the time of the submission of the manuscript. We now added supplementary figure 4 with images of a double lof mutant and added a sentence in lines 182-183 in the main text indicating that nhr-1 is epistatic.

(3) The analysis of potential targets of nhr-1 and nhr-40 forms a critical part of the paper, and is largely solid. However, a few issues could be cleared up. First, it would be helpful for them to emphasize clearly throughout that the targets could be direct or indirect. Second, have they tested any of the transcriptional reporters in nhr-1 or nhr-40 backgrounds? Looking at a few of these could give a clearer impression of cell-by-cell regulation, as opposed to the whole body regulation of the RNAseq studies.

Following the reviewer’s suggestion, we added a statement in lines 251-253 that the regulation of the identified targets by both NHRs may be indirect. As for testing the reporters in different mutant backgrounds, we believe it would not clarify the function of NHR-1 and NHR-40 in different cells, as all the reporters are expressed in the same single cell. Still, we tested one of the reporters in *nhr* mutants and observed no obvious change in the expression pattern.

(4) Perhaps most important, the knockouts of potential target genes were beautifully done, but the lack of phenotypes is surprising. Have the authors considered ablating the cell they implicate through the expression studies, g1D? This might be a quick and easy test to confirm that the central topic of half their paper is indeed involved in controlling mouth morph development.

This is a great point. We did attempt to laser-ablate g1D in juveniles and examine the phenotype in adult. However, this has proven to be challenging, as the cell is really large. First, we tried ablation with high laser intensity and high number of shots, but the developing juveniles displayed multiple abnormalities, and the survival rate was extremely low, possibly because the ablation damaged non-target tissues surrounding the pharynx (e.g. the nervous system). Later, we performed ablation with a lower laser intensity in animals expressing one of the g1D-specific transgenes, but the expression of the transgene was often retained in adulthood, which suggests that the cell remained at least partially functional. Therefore, we fully agree with the reviewer that ablation of g1D would provide an important functional test of one of our hypotheses, but unfortunately, technical limitations precluded us from conducting a “clean” experiment with conclusive results.

(5) The extreme redundancy the authors observe can also be problematic from an evolutionary perspective, and they should expand their discussion to address this point.

We are not absolutely sure what the reviewer means by saying that the functional redundancy is “problematic from an evolutionary perspective”. If they mean the fact that such genes will not be efficiently selected for, we agree. Therefore, in the manuscript, we discuss the rapid evolutionary turnover of the NHR-1 and -40 targets in the context of faster evolution of enzyme-encoding genes compared to transcription factors.

(6) Although we like the author’s evolutionary model at the end, they state it a little too strongly. For example, the sentences: “We speculate that the striking co-expression of the target genes results from an ancient regulatory linkage between the NHRs and the promoters of the ancestral target genes. Such divergent evolutionary dynamics of transcription factors and their downstream targets might represent general features of GRNs” This summary neglects the possibility of these transcription factors capturing new promoters by mutation of target sites. Do we have any information about how complex NHR-1 or NHR-40 target sequences might be?

We agree with the reviewer and have removed the last sentence of this statement. To indicate potential alternative scenarios, we instead, have added a sentence along the lines of the reviewer`s argument.

(7) Since all of the target genes are expressed in the pharyngeal gland cell, g1D, which has a long process that terminates into the buccal cavity, is it possible that the NHR target genes are involved in sensing environmental factors. For example, is the duodecuple Astacin mutant less sensitive to environmental signals.

We are not aware of any evidence that pharyngeal gland cells in nematodes perform environmental sensing, and ultrastructural observations are consistent with a strictly excretory role of g1D. Also, the duodecuple Astacin mutant has surprisingly, no obvious phenotype.

Minor Changes

Figure 2 FPKM should be defined in the legend

Done.

Line 98 Change “and we are yet to find” to “and we had yet to find”

Done.

Line 254 Change “NHR-40 and NHR-1 where more highly expressed” to “NHR-40 and NHR-1 were more highly expressed”

Done.

Line 390 Change “On the contrary, we identified” to “By contrast, we showed ”

Done.

**Reviewer #3:**

The only minor thing I have is that I find the section in page 14 lines 271-283 a bit speculative about what the targets might be doing based on their GO terms. The authors also tried hard to obtain phenotypes from the Astacin, chitinase, and CAP mutants without any luck. The authors explain this negative result by redundancy, which seems quite reasonable to me. I wonder if the authors observed any increase in expression of other members of Astacin, CAP, and chitinase in these mutants? This would confirm redundancy being the source for the lack of phenotypes.

We agree with the reviewer and have therefore shortened and downplayed the wording of this section, which is now starts in line 291. We were unable to perform the experiment suggested by the reviewer, because the generation of the duodecuple mutant took more than a year, and the PhD student, who is the first author of the study, had run out of time to do such a follow up study. Besides, transcriptional compensation is only one of the mechanisms of how functional redundancy may be achieved, hence it would be unclear how to interpret the negative result.

Taken together, we feel that have addressed all issues that the reviewers brought up.

Thank you very much for considering this revised manuscript.

Sincerely yours,

Ralf J. Sommer (on behalf of all co-authors).
